# Factors Associated With Mortality in Diffuse Large B‐Cell Lymphoma (DLBCL) With Disseminated Intravascular Coagulation (DIC): A National Inpatient Sample Analysis

**DOI:** 10.1155/ah/8956944

**Published:** 2026-08-16

**Authors:** Safia Ansari, Kristy Bono, Berk Madendere, Anand Shah, Joshua Kra

**Affiliations:** ^1^ Rutgers New Jersey Medical School, 150 Bergan St, Newark 07103, New Jersey, USA, rutgers.edu; ^2^ Rutgers Cancer Institute, 150 Bergan St, Newark 07103, New Jersey, USA

## Abstract

Diffuse large B‐cell lymphoma (DLBCL) is the most common subtype of non‐Hodgkin lymphoma. Hematologic malignancies are known precipitants of disseminated intravascular coagulation (DIC), and the coexistence of these conditions is associated with poor outcomes; however, large‐scale data specifically examining concurrent DLBCL and DIC are limited. We performed a retrospective cohort study using the National Inpatient Sample (NIS) to evaluate in‐hospital mortality among adult hospitalizations with DIC and/or DLBCL identified using ICD‐10 codes. Adults aged ≥ 18 years with a diagnosis of DLBCL and/or DIC were included. In‐hospital mortality among patients with concurrent DLBCL and DIC was 54.1%, significantly higher than mortality observed in patients with either condition alone (*p* < 0.001). In multivariable analysis restricted to patients with both DLBCL and DIC, independent predictors of increased mortality included HIV/AIDS (aOR 4.266, 95% CI: 1.886–9.649; *p* < 0.001), septic shock (aOR 3.201, 95% CI: 2.305–4.443; *p* < 0.001), mechanical ventilation (aOR 2.948, 95% CI: 2.175–3.995; *p* < 0.001), vasopressor use (aOR 2.759, 95% CI: 1.789–4.255; *p* < 0.001), chronic kidney disease (aOR 2.523, 95% CI: 1.596–3.989; *p* < 0.001), acute kidney injury (aOR 1.884, 95% CI: 1.347–2.637; *p* < 0.001), Black race (aOR 2.221, 95% CI: 1.418–3.477; *p* < 0.001), and Asian or Pacific Islander race (aOR 2.383, 95% CI: 1.329–4.271; *p* = 0.004) compared to White patients. Bleeding events, thromboembolic complications, stem cell transplantation, and CAR‐T therapy were not independently associated with mortality. These findings demonstrate that among patients with DLBCL, the development of DIC is associated with markedly elevated in‐hospital mortality, driven predominantly by critical illness, organ dysfunction, and comorbidity burden rather than bleeding or thrombosis alone. Racial disparities in outcomes were independently observed after adjusting for illness severity and comorbidities, warranting further investigation. Early recognition of DIC in patients with DLBCL and aggressive management of systemic complications, particularly septic shock, respiratory failure, and renal dysfunction, may help mitigate disease severity and improve outcomes.

## 1. Introduction

Diffuse large B‐cell lymphoma (DLBCL) is the most common subtype of non‐Hodgkin lymphoma (NHL), representing 25%–40% of all malignant lymphoid neoplasms [1–3]. There are several risk factors associated with the development of DLBCL, including HIV/AIDS, inherited immunodeficiency syndromes, organ transplant, environmental exposures, obesity, and viruses such as Hepatitis B virus [4]. The clinical presentation of DLBCL is variable depending on the extranodal site, and it is overall aggressive yet treatable. With contemporary treatment strategies, including a combination of include chemotherapy, radiotherapy, and immunomodulating agents, cure rates range from 50% in patients with advanced disease and 80%–85% in patients with limited disease [5].

Disseminated intravascular coagulation (DIC) is an acquired, life‐threatening syndrome characterized by activation of coagulation pathways, leading to widespread microvascular thrombosis, consumption of platelets and coagulation factors, and subsequent bleeding and organ dysfunction. DIC can originate from a multitude of factors, including infection, hematologic malignancies, obstetric diseases, trauma, or vascular disorders [6]. The clinical presentation is highly variable and can be a diagnostic challenge. Patients can present with both bleeding and thrombosis, with clot formation primarily occurring in the microvasculature and manifesting as organ failure, such as renal and hepatic dysfunction, lung injury, and cerebral dysfunction [7]. Activation of coagulation systems can lead to a consumptive coagulopathy and present as bleeding in the form of petechiae, ecchymosis, gastrointestinal bleeds, respiratory bleeds, and bleeding from surgical sites [6, 7]. DIC has a poor prognosis with a 30‐day mortality rate of 20%–45% [7–9].

Hematologic malignancies, including NHL, are known to precipitate DIC [10, 11]. Previous studies evaluating DIC in NHL populations have demonstrated that the development of DIC exerts a significantly negative impact on the prognosis of NHL [12]. However, there are limited data on DIC on patients with DLBCL on a large scale population. This study aims to assess the risk of inpatient mortality in patients with DIC and/or DLBCL using data from the National Inpatient Sample (NIS). The primary objective of the study is to determine a mortality risk in patients with DIC and DLBCL. Secondary objectives included identifying demographic, clinical, and comorbidity‐related predictors of mortality in this high‐risk population.

## 2. Methods

### 2.1. Data Source

We conducted a retrospective cohort study using the Healthcare Cost and Utilization Project (HCUP) NIS from 2016 to 2019. The NIS is the largest all‐payer inpatient database in the United States and represents approximately 20% of the hospital discharges, weighted to produce national estimates. It contains deidentified patient‐level data including demographics, diagnoses, procedures, hospital characteristics, outcomes, and resource utilization. Because the NIS contains publicly available, deidentified data, this study was exempt from institutional review board review.

### 2.2. Study Population

The analytic cohort consisted of adult patients (≥ 18 years) hospitalized with a diagnosis of DLBCL and/or DIC. Patients without either diagnosis were excluded. Diagnoses were identified using ICD‐10‐CM diagnosis codes (see Supporting Table 2: Diseases and corresponding ICD‐10‐CM codes for Data Analysis). Each hospitalization was treated as an independent observation.

As an initial step, two complementary unadjusted analyses were performed using chi‐square testing: (1) Among hospitalizations with DIC, in‐hospital mortality was compared between patients with vs without DLBCL. (2) Among hospitalizations with DLBCL, in‐hospital mortality was compared between patients with vs. without DIC. These analyses were conducted on separately filtered datasets, restricted to DIC‐positive and DLBCL‐positive cohorts, respectively. Using results from these analyses, patients were categorized into three mutually exclusive groups: DLBCL only, DIC only, and DLBCL + DIC.

Following this comparison, subsequent analyses were restricted to patients with concurrent DLBCL and DIC to identify predictors of mortality within this high‐risk subgroup. Patients with missing mortality data were excluded. The final analytic cohort consisted of patients with complete data for outcomes and covariates.

### 2.3. Outcomes

The primary outcome was in‐hospital mortality, defined as death occurring during the index hospitalization as recorded in the discharge disposition field of the NIS. Secondary outcomes included identification of demographic, clinical, and comorbidity‐related predictors of in‐hospital mortality among patients with concurrent DLBCL and DIC.

### 2.4. Exposure and Covariates

Markers of acute critical illness and organ dysfunction during hospitalization were identified using ICD‐10‐CM diagnosis and procedure codes and included septic shock, mechanical ventilation, vasopressor use, acute kidney injury (AKI), acute respiratory failure (ARF), major bleeding, pulmonary embolism (PE), deep vein thrombosis (DVT), acute respiratory distress syndrome (ARDS), and dialysis. All variables were modeled as binary indicators (present vs. absent during hospitalization).

Baseline comorbidity burden was quantified using the Charlson Comorbidity Index (CCI), calculated from ICD‐10‐CM diagnosis codes (See Supporting Table 1: Diseases and corresponding ICD‐10‐CM codes used to calculate the CCI). Individual Charlson comorbidities were identified and weighted according to the validated Charlson scoring system. The total CCI score was treated as a continuous variable in regression analyses.

Demographic covariates included age at admission, modeled as a continuous variable, race, categorized using standardized database groupings, and primary expected payer, categorized as Medicare, Medicaid, private insurance, self‐pay, or other.

### 2.5. Statistical Analysis

Continuous variables were summarized using means and standard deviations, while categorical variables were summarized using counts and percentages.

Prior to multivariable modeling, collinearity between key covariates—including CCI, AKI, pulmonary disease, and age—was assessed using Pearson correlation coefficients. Correlation coefficients < 0.3 were considered indicative of low collinearity and acceptable for inclusion in multivariable models. No variables met criteria for exclusion based on collinearity.

Multivariable logistic regression was used to identify independent predictors of in‐hospital mortality among patients with concurrent DLBCL and DIC. Variables were selected a priori based on clinical relevance and biological plausibility, including markers of acute organ dysfunction, critical care interventions, baseline comorbidity burden, and age.

The final multivariable model included mechanical ventilation, septic shock, vasopressor use, AKI, acute renal failure, CCI, and age at admission.

Model fit was assessed using the omnibus test of model coefficients and pseudo‐*R* ^2^ statistics (Cox & Snell and Nagelkerke *R* ^2^). Model discrimination was evaluated using classification tables with a cutoff probability of 0.50. Adjusted odds ratios (ORs) with 95% confidence intervals (CIs) are reported. A two‐sided *p* value < 0.05 was considered statistically significant.

## 3. Results

### 3.1. Study Population

A total of 436,125 adult hospitalizations were included in this study. A total of 159,005 patients with a diagnosis of DIC were identified. Additionally, 276,010 patients with a diagnosis of DLBCL were included. A total of 1110 patients had a concurrent diagnosis of DIC and DLBCL. All hospitalizations had complete data for in‐hospital mortality and key covariates and were included in the analysis.

### 3.2. In‐Hospital Mortality by DLBCL and DIC Statuses

Among hospitalizations with DLBCL, patients with concurrent DIC experienced markedly higher mortality compared with those without DIC (54.1% vs. 4.3%, *p* < 0.001). This remained significant after adjustment for demographic factors such as age, race, and insurance status.

Additionally, among hospitalizations with DIC, patients with concurrent DLBCL had significantly higher in‐hospital mortality compared with those without DLBCL (54.1% vs. 49.0%, *p* < 0.001). This also remained significant after adjustment.

**FIGURE 1:**
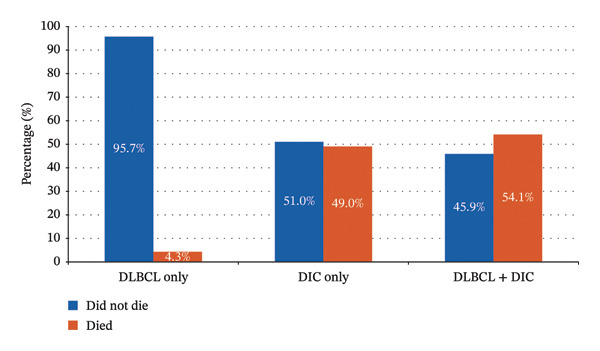
In‐hospital mortality by DLBCL and DIC statuses.

Mortality was highest among patients with concurrent DLBCL and DIC, exceeding that observed in either condition alone (*χ* ^2^ *p* < 0.001) (see Table 1 and Figure 1).

**TABLE 1:** In‐hospital mortality by DLBCL and DIC statuses.

Group	DLBCL	DIC	Did not die, (%)	Died, (%)	Total
DLBCL only	Yes	No	264,085 (95.7%)	11,925 (4.3%)	276,010
DIC only	No	Yes	81,160 (51.0%)	77,845 (49.0%)	159,005
DLBCL + DIC	Yes	Yes	510 (45.9%)	600 (54.1%)	1110
Total					436,125

### 3.3. Characteristics of Patients With Concurrent DLBCL and DIC

Subsequent analyses focused on a subset of patients with concurrent DLBCL and DIC. A total of 1110 patients were included in this subset. The mean age at admission was 62 years, 55.4% were male, and 44.6% were female. A total of 59% of the patients were White, 15.8% were Black, 14% were Hispanic, 5.4% were Asian, and 5% were other (see Table 2).

**TABLE 2:** Sociodemographic characteristics of patients with DLBCL and DIC.

Characteristic	Did not die	Died	Total	Significance
Sex				*p* = 0.756
Male	280 (45.5%)	335 (54.5%)	615	
Female	230 (46.5%)	265 (53.5%)	495	
Race				*p* < 0.001
White	330 (49.6%)	335 (50.4%)	665	
Black	55 (31.4%)	120 (68.6%)	175	
Hispanic	75 (48.4%)	80 (51.6%)	155	
Asian/Pacific Islander	25 (41.7%)	35 (58.3%)	60	
Other	25 (45.5%)	30 (54.5%)	55	
Primary payor				*p* = 0.252
Medicare	235 (46.5%)	270 (53.5%)	505	
Medicaid	85 (40.5%)	125 (59.5%)	210	
Private insurance	160 (49.2%)	165 (50.8%)	325	
Self‐pay	15 (37.5%)	25 (62.5%)	40	
Other	15 (50.0%)	15 (50.0%)	30	
Hospital region				*p* < 0.001
“Central” counties of metro areas of ≥ 1 million population	185 (41.1%)	265 (58.9%)	450	
“Fringe” counties of metro areas of ≥ 1 million population	130 (47.3%)	145 (52.7%)	275	
Counties in metro areas of 250,000–999,999 population	105 (55.3%)	85 (44.7%)	190	
Counties in metro areas of 50,000–249,999 population	55 (64.7%)	30 (35.3%)	85	
Micropolitan counties	20 (33.3%)	40 (66.7%)	60	
Not metropolitan or micropolitan counties	15 (30.0%)	35 (70.0%)	50	

The mean CCI score was 8.1, reflecting a high baseline comorbidity burden. Pearson correlation analysis demonstrated low correlations between the CCI and key acute illness variables, including AKI (*r* = 0.159) and pulmonary disease (*r* = 0.116). No correlations exceeded thresholds suggestive of problematic collinearity, supporting inclusion of CCI and acute organ dysfunction variables within the same multivariable model. In raw analyses, motality in this subset of patients was positively correleated with several comorbid conditions and complications (Table 3).

**TABLE 3:** Comorbidities and complications in patients with concurrent DLBCL and DIC.

Predictor	Did not die	Died	Total	Significance
Acute kidney injury	305 (37.9%)	500 (62.1%)	805	*p* < 0.001
Acute respiratory distress syndrome	0 (0%)	5 (100%)	5	*p* = 0.046
Acute respiratory failure	145 (28.4%)	365 (71.6%)	510	*p* < 0.001
Arrhythmia	50 (35.7%)	90 (64.3%)	140	*p* = 0.006
Blood transfusion	210 (45.7%)	250 (54.3%)	460	*p* = 0.459
Cardiogenic shock	10 (28.6%)	25 (71.4%)	35	*p* < 0.001
CAR‐T therapy	10 (40.0%)	15 (60.0%)	25	*p* = 0.347
Chronic kidney disease	40 (27.6%)	105 (72.4%)	145	*p* < 0.001
Congestive heart failure	85 (42.5%)	115 (57.5%)	200	*p* = 0.158
Diabetes mellitus	130 (53.1%)	115 (46.9%)	245	*p* = 0.007
Dialysis	60 (38.7%)	95 (61.3%)	155	*p* = 0.031
GI bleed	50 (50.0%)	50 (50.0%)	100	*p* = 0.227
HIV/AIDS	10 (20.0%)	40 (80%)	50	*p* < 0.001
Hypertension	150 (54.5%)	125 (45.5%)	275	*p* < 0.001
Major bleed	30 (45.9%)	40 (57.1%)	70	*p* = 0.341
Mechanical ventilation	125 (24.5%)	385 (75.5%)	510	*p* < 0.001
Myocardial infarction	25 (31.3%)	55 (68.8%)	80	*p* = 0.004
Pneumonia	145 (42.0%)	200 (58.0%)	345	*p* = 0.045
Pulmonary embolism	20 (44.4%)	25 (55.6%)	45	*p* = 0.480
Sepsis	210 (32.3%)	440 (67.7%)	650	*p* < 0.001
Septic shock	140 (25.9%)	400 (74.1%)	540	*p* < 0.001
Stem cell transplant	15 (42.9%)	20 (57.1%)	35	*p* = 0.442
Stroke	25 (62.5%)	15 (37.5%)	40	*p* = 0.024
Tobacco use	105 (46.7%)	120 (53.3%)	225	*p* = 0.433
Vasopressor use	35 (17.9%)	160 (82.1%)	195	*p* < 0.001

### 3.4. Multivariable Predictors of In‐Hospital Mortality

In multivariable logistic regression analysis restricted to patients with concurrent DLBCL and DIC, several factors were independently associated with increased odds of in‐hospital mortality (Table 4).

**TABLE 4:** Multivariate logistic regression for patients with concurrent DLBCL and DIC.

Predictor	Adjusted OR	95% CI	*p* value
Age	1.008	1.002–1.024	0.132
Race (overall)			< 0.001
White			
Black	2.221	1.418–3.477	< 0.001
Hispanic	0.716	0.462–1.108	0.134
Asian/Pacific Islander	2.383	1.329–4.271	0.004
Other race	1.390	0.707–2.731	0.340
Mechanical ventilation	2.948	2.175–3.995	< 0.001
Septic shock	3.201	2.305–4.443	< 0.001
Vasopressor use	2.759	1.789–4.255	< 0.001
Acute kidney injury	1.884	1.347–2.637	< 0.001
Chronic kidney disease	2.523	1.596–3.989	< 0.001
HIV/AIDS	4.266	1.886–9.649	< 0.001
Charlson Comorbidity Index	1.020	0.977–1.066	0.363

**FIGURE 2:**
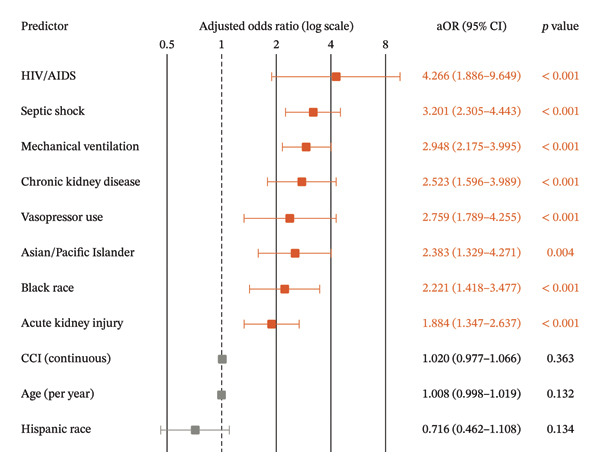
Multivariable predictors of in‐hospital mortality (DLBCL + DIC).

The majority of patients with DLBCL and DIC had sepsis (58.6%). A significant number of patients had septic shock (48.6%), AKI (72.5%), and mechanical ventilation (45.9%). All of these factors were strongly and independently associated with increased mortality—most strongly septic shock (aOR 3.201, 95% CI: 2.305–4.443; *p* < 0.001), followed by mechanical ventilation (aOR 2.948, 95% CI: 2.175–3.995; *p* < 0.001) and AKI (aOR 1.884, 95% CI: 1.347–2.637; *p* < 0.001). Additionally, vasopressor use was found in a smaller percentage of patients (17.6%) but was strongly associated with increased mortality (aOR 2.759, 95% CI: 1.789–4.255; *p* < 0.001) (see Figure 2).

Race was significantly associated with mortality in patients with DLBCL and DIC (*p* < 0.001), even after adjusting for comorbidities and illness severity. Compared to White patients, Black patients had significantly higher odds of in‐hospital death (aOR 2.221, 95% CI: 1.418–3.477; *p* < 0.001), as did Asian or Pacific Islander patients (aOR 2.383, 95% CI: 1.329–4.271; *p* = 0.004). Hispanic race (aOR 0.716, 95% CI: 0.462–1.108; *p* = 0.134) and other race (aOR 1.390, 95% CI: 0.707–2.731; *p* = 0.340) were not significantly associated with differential mortality compared to White patients.

Among pre‐existing comorbidities, chronic kidney disease was associated with approximately 2.5‐fold increased odds of in‐hospital death (aOR 2.523, 95% CI: 1.596–3.989; *p* < 0.001). HIV/AIDS demonstrated one of the strongest independent associations with mortality in the cohort (aOR 4.266, 95% CI: 1.886–9.649; *p* < 0.001).

Although 41.4% of the patients required blood transfusions, this was not associated with a higher mortality. Likewise, major bleeding, blood transfusions, PE, DVT, ARDS, tobacco use, congestive heart failure, chimeric antigen receptor (CAR)‐T therapy, stem cell transplant (SCT), and patient sex were not associated with increased mortality in the original analysis and were excluded from subsequent analyses. Variables including myocardial infarction, cardiogenic shock, pneumonia, ARDS, dialysis, hypertension, diabetes mellitus, arrhythmia, age, primary payor, and hospital region were not independently associated with mortality after adjustments and were excluded from the final model. Additionally, stroke was associated with decreased mortality; however, this represented a small patient sample (*n* = 40) and is unlikely of clinical significance.

## 4. Discussion

In patients with DLBCL, the development of DIC was associated with markedly elevated in‐hospital mortality, with a crude mortality rate of 54.1% among those with concurrent disease compared to 4.3% among those with DLBCL alone. We identified several independent predictors of mortality, including race, markers of critical illness, and comorbid conditions.

Hematologic malignancies, including NHL, are well‐established precipitants of DIC, and the presence of DIC in this population has consistently been associated with poor outcomes and high mortality [10]. However, data evaluating the specific factors that drive mortality among patients with concurrent DIC and DLBCL remain limited. Using a large, nationally representative inpatient database, we demonstrate a strong bidirectional association between DIC and DLBCL with in‐hospital mortality. Patients with both DIC and DLBCL experienced significantly higher mortality compared with those with either condition alone (54.1% vs. 4.3% and 49.0%, respectively; *p* < 0.001).

Sepsis and septic shock were both strongly associated with increased inpatient mortality in patients with DIC and DLBCL. Patients with NHL are at high risk for the development of secondary immunodeficiency and secondary immunodeficiency–related infections and mortality. This is driven by a combination of the malignancy itself and cancer treatments, as well as comorbid conditions that can be aggravated by cancer treatments [13]. In parallel, sepsis‐associated DIC itself is a highly fatal condition. The pathophysiology of DIC in patients with sepsis is multifactorial, including increase in platelet reactivity from bacterial endotoxins, increased platelet reactivity from increased hormone levels, and increased thrombin generation by the coagulation cascade [14].

In our cohort of patients with both DLBCL and DIC, sepsis was present in 41% of the patients who survived hospitalization and in 73% of those who died, suggesting that sepsis is a major contributing factor to both the development of DIC and subsequent mortality. On multivariable analysis, septic shock was the strongest independent predictor of death (aOR 3.201, 95% CI: 2.305–4.443; *p* < 0.001), followed by mechanical ventilation (aOR 2.948, 95% CI: 2.175–3.995; *p* < 0.001) and vasopressor use (aOR 2.759, 95% CI: 1.789–4.255; *p* < 0.001). Early recognition of infection, aggressive antimicrobial therapy, and close monitoring for evolving coagulopathy may, therefore, represent critical opportunities for intervention.

Prior studies have demonstrated that the SCT and the more recently introduced CAR‐T‐cell therapy can lead to coagulopathy and ultimately DIC [10]. Additionally, both SCT and CAR‐T therapy have been linked to increased infection, sepsis, and death [15, 16]. However, in our cohort, only a small percentage underwent STC or CAR‐T therapy, and neither exposure was associated with increased mortality. Similarly, bleeding is often cited as a major cause of mortality in patients with hematological cancer–associated DIC [17]. Yet, in this study, neither bleeding nor embolic event were associated with increased mortality. Instead, factors such as mechanical ventilation, vasopressor use, and AKI were independently associated with increased mortality. These findings suggest that in inpatient settings, mortality in DIC complicating DLBCL is driven less by isolated hemorrhagic or thrombotic events and more by multiorgan failure in the context of severe systemic inflammation and shock.

Our analysis identified HIV/AIDS as one of the strongest independent predictors of mortality, associated with a more than fourfold increase in the odds of in‐hospital death (aOR 4.266, 95% CI: 1.886–9.649; *p* < 0.001). Patients with HIV are at elevated risk of developing lymphomas due to severe immunosuppression, and NHLs represent the most common HIV‐associated malignancy and a leading cause of death in this population in developed countries [18]. Additionally, HIV infection is associated with baseline dysregulation of the coagulation system, which may predispose affected patients to develop DIC [19]. Importantly, HIV remained independently associated with increased mortality after controlling for sepsis and other markers of critical illness, suggesting that the excess risk is not solely mediated through infection. This finding highlights HIV infection as a uniquely vulnerable clinical context in which the coexistence of lymphoma and DIC portends particularly poor outcomes and warrants heightened clinical vigilance.

Racial disparities in mortality were also independently observed in this cohort. Compared to White patients, Black patients (aOR 2.221, 95% CI: 1.418–3.477; *p* < 0.001) and Asian or Pacific Islander patients (aOR 2.383, 95% CI: 1.329–4.271; *p* = 0.004) had significantly higher odds of in‐hospital death after adjusting for comorbidities and markers of illness severity. Hispanic and other race were not significantly associated with differential mortality. The mechanisms underlying these disparities are likely multifactorial and may reflect differences in access to care, disease presentation at time of hospitalization, institutional resource availability, or unmeasured socioeconomic factors [20, 21]. These findings warrant further investigation to identify modifiable contributors to racial inequity in outcomes among this high‐risk population.

This study has several limitations inherent to retrospective analyses of administrative databases. Diagnoses were identified using ICD‐10 codes, which may be subject to misclassification or undercoding. Detailed clinical variables such as laboratory markers of coagulopathy, timing of DIC onset, disease stage, specific chemotherapy regimens, and performance status were unavailable. Unfortunately, since the time of onset of either condition is unknown, a temporal relationship between DLBCL and DIC onset cannot be established. Specifically, chemotherapy‐induced tumor lysis syndrome (TLS) is a recognized precipitant of DIC and multiorgan failure that could not be captured or adjusted for in this analysis, representing an important unmeasured confounder. Causal relationships cannot be inferred due to the observational nature of the study. Finally, the NIS captures only inpatient encounters, precluding the assessment of postdischarge outcomes.

## 5. Conclusion

Concurrent DIC and DLBCL are associated with markedly increased inpatient mortality, driven primarily by sepsis, multiorgan failure, and underlying immunosuppression rather than hemorrhagic or thrombotic complications alone. HIV infection and Black and Asian or Pacific Islander race were independently associated with worse outcomes after adjusting for illness severity and comorbidity burden. These findings underscore the importance of early infection control, aggressive supportive care, and targeted risk stratification in patients with DLBCL complicated by DIC and highlight the need for further investigation into racial disparities in this population.

## Author Contributions

Safia Ansari: conceptualization, methodology, data curation, formal analysis, investigation, writing–original draft, and writing–review and editing.

Kristy Bono: investigation, data curation, and review and editing.

Berk Madendere: investigation, data curation, and review and editing.

Anand Shah: investigation, data curation, and review and editing.

Joshua Kra: conceptualization, supervision, methodology, project administration, and writing–review and editing.

## Funding

This research received no external funding.

## Disclosure

All authors have read and approved the final version of the manuscript. Safia Ansari, DO, had full access to all of the data in this study and takes complete responsibility for the integrity of the data and the accuracy of the data analysis.

## Conflicts of Interest

The authors declare no conflicts of interest.

## Supporting Information

Additional supporting information can be found online in the Supporting Information section.

## Supporting information


**Supporting Information** Supporting Table 1: Diseases and corresponding ICD‐10‐CM codes used to calculate the Charlson Comorbidity Index (CCI) include diseases and corresponding ICD‐10 codes for CCI. Supporting Table 2: Diseases and corresponding ICD‐10‐CM codes for Data Analysis include all other disease and procedure codes used throughout the manuscript.

## Data Availability

All data supporting this work were taken from the Healthcare Cost and Utilization Project (HCUP) National Inpatient Sample (NIS) database: https://hcup-us.ahrq.gov/db/nation/nis/nisdbdocumentation.jsp.
