# Supplementary material for: Factors Associated With Mortality in Diffuse Large B‐Cell Lymphoma (DLBCL) With Disseminated Intravascular Coagulation (DIC): A National Inpatient Sample Analysis
Source: Adv Hematol. 2026 Aug 16;2026:8956944. doi: 10.1155/ah/8956944 (PMC13478723; doi:10.1155/ah/8956944)
Supplement: Supplementary file 1 [file AH-2026-8956944-s001.docx]

**Supplementary Table 1: Diseases and corresponding ICD-10-CM codes used to calculate the Charlston Comorbidity Index (CCI)**

| **Disease** | **ICD-10-CM Code** | **CCI Score** |
| --- | --- | --- |
| Diabetes mellitus | E10-E14 | 1 |
| Myocardial Infarction | I21, I22, I252 | 1 |
| Congestive Heart Failure | I50 | 1 |
| Peripheral Vascular Disease | I70-I79 | 1 |
| Cerebrovascular Disease | I60-I69 | 1 |
| Dementia | F01-F03, G30, G310, F04, F05, F061, F068, G132, G138, G311, G312, R4181 | 1 |
| Chronic Pulmonary Disease | J41-J45, J47, J60-J67, J701, J703 | 1 |
| Rheumatic or Connective Tissue Disease | M30-M36, M05, M06 | 1 |
| Gastric or Peptic Ulcer | K25, K26 | 1 |
| Mild Liver Disease | B18, B19, K70-K77 | 1 |
| Hemiplegia or Paraplegia | G81-G83, G041, G114, G800, G801, G802 | 2 |
| Moderate-Severe Liver Disease | I850, K704, K711, K721, K729, I864, K765, K766, K767 | 2 |
| Any Malignancy, except BCC of skin | C00-C41, C43, C45-C72, C74, C75, C76, C81-C84, C85 | 2 |
| Metastatic Solid Tumor | C76-C79, C800, C802 | 6 |
| Acquired Immune Deficiency Syndrome | B20-B24 | 6 |

**Supplementary Table 2: Diseases and corresponding ICD-10-CM codes for Data Analysis**

| DIC | D65 |
| --- | --- |
| DLBCL | C83.30, C83.31, C83.32, C83.33, C83.34, C83.35, C83.36, C83.37, C83.38, C83.39 |
| HIV/AIDS | B20  Z21 |
| Chronic Kidney Disease | N18.3 N 18.30, N18.31, N18.32,  N18.4,  N18.5, N18.6, Z99.2, 5A1D70Z, 5A1D80Z, 5A1D90Z, 3E1M39Z |
| Tobacco Use | F17200, F17201, F17203, F17208, F17209, F17210, F17211, F17213, F17218, F17219, Z720, Z87891 |
| Arrythmias | I4901; I472; I480; I481; I482; I483; I484; I491; I492 |
| Major bleeding | R58, L7622, K661, I62, I620, I6200, I6201, I6202, I6203, I621, I629, R04, R040, R041, R042, R048, R0481, R0489, R049, N938, N939 |
| Gastrointestinal bleeding | K920, K921, K922, K625, K928, K929, K9281, K9289, K250, K254, K260, K262, K270, K28, K621 |
| Acute Kidney Injury | N170, N171, N172, N178, N179 |
| Pulmonary embolism | I26; I260, I2602, I2609, I269, I 2692, I2699  I2602, I2609, I2690, I2692, I2693, I2694, I2699 |
| Sepsis | A021, A20.7, A22.7, A26.7, A32.7, A40, A41, A41.0, A41.01, A41.02, A41.3, A41.4, A41.5, A41.51, A41.52, A41.53, A41.53, A41.59, A41.8, A41.81, A41.89, A41.9, A54.86, T81.4 |
| Septic shock | R65.21 |
| Cardiogenic shock | R570 |
| Blood transfusion | 30230H0, 30230H1, 30230J0, 30230J1, 30230K0, 30230K1, 30230L0, 30230L1, 30230M0, 30230M1, 30230N0, 30230N1, 30230P0, 30230P1, 30230Q0, 30230Q1, 30230R0, 30230R1, 30230S0, 30230S1, 30230T0, 30230T1, 30230V0, 30230V1, 30230W0, 30230W1, 30233H0, 30233H1, 30233J0, 30233J1, 30233K0, 30233K1, 30233L0, 30233L1, 30233M0, 30233M1, 30233N0, 30233N1, 30233P0, 30233P1, 30233Q0 ,30233Q1, 30233R0, 30233R1, 30233S0, 30233S1, 30233T0, 30233T1, 30233V0, 30233V1, 30233W0, 30233W1, 30240H0, 30240H1, 30240J0, 30240J1, 30240K0, 30240K, 30240L0, 30240L1, 30240M0, 30240M1, 30240N0, 30240N1, 30240P0, 30240P1, 30240Q0, 30240Q1, 30240R0, 30240R1, 30240S0, 30240S1, 30240T0, 30240T1, 30240V0, 30240V1, 30240W0, 30240W1, 30243H0, 30243H1, 30243J0, 30243J1, 30243K0, 30243K1, 30243L0, 30243L1, 30243M0, 30243M1, 30243N0, 30243N1, 30243P0, 30243P1, 30243Q0, 30243Q1, 30243R0, 30243R1, 30243S0, 30243S1, 30243T0, 30243T1, 30243V0, 30243V1, 30243W0, 30243W1, 30250H0, 30250H1, 30250J0, 30250J1, 30250K0, 30250K1, 30250L0, 30250L1, 30250M0, 30250M1, 30250N0, 30250N1, 30250P0, 30250P1, 30250Q0, 30250Q1, 30250R0, 30250R1, 30250S0, 30250S1, 30250T0, 30250T1, 30250V0, 30250V1, 30250W0, 30250W1, 30253H0, 30253H1, 30253J0, 30253J1, 30253K0, 30253K1, 30253L0, 30253L1, 30253M0, 30253M1, 30253N0, 30253N1, 30253P0, 30253P1, 30253Q0, 30253Q1, 30253R0, 30253R1 , 30253S0, 30253S1, 30253T0, 30253T1, 30253V0, 30253V1, 30253W0, 30253W1, 30260H0, 30260H1, 30260J0, 30260J1, 30260K0, 30260K1, 30260L0, 30260L1, 30260M0, 30260M1, 30260N0, 30260N1, 30260P0, 30260P1, 30260Q0, 30260Q1, 30260R0, 30260R1, 30260S0, 30260S1, 30260T0,30260T1, 30260V0, 30260V1, 30260W0, 30260W1, 30263H0, 30263H1, 30263J0, 30263J1, 30263K0, 30263K1 30263L0, 30263L1, 30263M0, 30263M1, 30263N0, 30263N1, 30263P0, 30263P1, 30263Q0, 30263Q1, 30263R0, 30263R1, 30263S0, 30263S1, 30263T0, 30263T1, 30263V0, 30263V1, 30263W0, 30263W1, 30273H0, 30273H1, 30273J0, 30273J1, 30273K0, 30273K1, 30273L0 30273L1,  30273M0, 30273M1, 30273N0, 30273N1, 30273P0, 30273P1, 30273Q0, 30273Q1 30273R0, 30273R1, 30273S0, 30273S1, 30273T0, 30273T1, 30273V0, 30273V1, 30273W0 30273W1, 30277H1, 30277J1, 30277K1, 30277L1, 30277M1, 30277N1 30277P1, 30277Q1, 30277R1, 30277S1, 30277T1, 30277V1, 30277W1, 30280B1, 30283B1, 3E030GC ,3E033GC 3E040GC, 3E043GC ,3E050GC ,3E053GC ,3E060GC, 3E063GC. |
| Vasopressor | 3E050XZ; 3E063XZ; 3E060XZ; 3E053XZ; 3E043XZ; 3E040XZ; 3E033XZ; 3E030XZ |
| Mechanical ventilation | 5A1935Z, 5A1945Z, 5A1955Z |
| Dialysis | 5A1D00Z, 5A1D60Z, 5A1D70Z |
| CART Therapy | XW033C3  XW043C3 |
| Stem Cell Transplant | Z9481;T8600;T8601;T8602;T8603;T8609;Z48290 |
